# Dietary Fiber and Cancer Management: A Twenty-Five-Year Bibliometric Review of Research Trends and Directions

**DOI:** 10.1155/bmri/5086946

**Published:** 2025-04-29

**Authors:** Aliu Moomin, Sidique Gawusu, Abubakar Ibn Sidik, Marizuk Waris Tizumah, Maridia Kunateh Adam, Paa Kofi Tawiah Adu-Gyamfi, Kwesi Boadu Mensah

**Affiliations:** ^1^Rowett Institute, University of Aberdeen, Aberdeen, Scotland, UK; ^2^Aberdeen Cancer Centre, University of Aberdeen, Aberdeen, Scotland, UK; ^3^Whiting School of Engineering, Johns Hopkins University, Baltimore, Maryland, USA; ^4^Department of Surgical Specialties, RUDN University, Moscow, Russia; ^5^Department of Medical laboratory/Medical Imaging Technology, Accra Technical University, Accra, Greater Accra, Ghana; ^6^Medical Centre, University of Ghana, Accra, Greater Accra, Ghana; ^7^Department of Data Science, University of East London, London, England, UK; ^8^Department of Dietetics, Robert Gordon University, Aberdeen, Scotland, UK; ^9^Department of Nursing and Midwifery, Pentecost University College, Accra, Greater Accra, Ghana; ^10^Department of Pharmacology, Kwame Nkrumah University of Science and Technology, Kumasi, Ashanti Region, Ghana

**Keywords:** bibliometric analysis, cancer management, chemotherapy, dietary fiber, immunotherapy, radiotherapy

## Abstract

**Background:** The global burden of cancer necessitates innovative approaches to its management and treatment. Traditional treatments like radiotherapy, immunotherapy, surgery, and chemotherapy, carry significant side effects that impact patient quality of life. Dietary fiber has attracted research interest as a potential mitigator of cancer progression and a supportive agent in cancer treatment. This bibliometric study analyzes trends in research connecting dietary fiber, cancer therapy, and gut health from April 1999 to May 2024.

**Methods and Results:** Web of Science, PubMed, and Scopus databases were used to retrieve peer-reviewed publications from April 1999 to May 2024 on dietary fiber and cancer management. The study identifies a rising scholarly interest in dietary fiber's role in cancer management, focusing significantly on the interactions between dietary fiber and gut microbiota. These interactions are particularly noted for their influence on inflammation and cancer metastasis. The study highlights evolving research themes, the importance of specific fiber types in cancer progression, and highlights persistent foundational themes like glycosylation. Emerging areas include personalized nutrition and innovative therapeutic approaches. The geographical and institutional contributions, mainly from the United States and China, underline the significance of collaborative and interdisciplinary efforts in advancing research.

**Conclusion:** This analysis emphasizes integrating dietary strategies in comprehensive cancer care and aims to address research gaps to develop more effective and patient-centered cancer therapy and prevention strategies.

**Summary**

• This study analyzes trends in research connecting dietary fiber, cancer therapy, and gut health from April 1999 to May 2024.
• The study identifies a rising scholarly interest in dietary fiber's role in cancer management, focusing significantly on the interactions between dietary fiber and gut microbiota.
• It emphasizes integrating dietary strategies in comprehensive cancer care.

## 1. Introduction

Cancer remains one of the most significant global health challenges, characterized by an array of diseases that can affect any part of the body, often with substantial individual and societal impact [1–3]. Cancer causes 2%–3% of deaths annually throughout the world which accounted for approximately 10 million deaths globally in 2018 [4]. Despite advances in medical science, cancer continues to be a leading cause of mortality worldwide, necessitating persistent efforts in research and healthcare innovation to manage and reduce its burden [5–8].

Current cancer treatments, including surgery, radiotherapy, chemotherapy, and immunotherapy, are the cornerstone of cancer care [9–11]. While these treatments can be effective, they are often associated with a host of side effects ranging from mild to severe, which can significantly impact patients' quality of life.

Several chemotherapy medications, such as 5-fluorouracil, anthracycline, and methotrexate, are utilized to treat various types of cancer, including breast, colon, lung, and stomach cancers and leukaemia [12]. Nevertheless, these medications are closely associated with severe adverse reactions, including fatigue, headache, alopecia, anorexia, heightened susceptibility to bleeding, thrombosis, dermatitis, gastrointestinal perforations, and secondary malignancies [12, 13].

Surgery is often contraindicated in elderly people due to age-related health conditions such as atheroma, elevated blood pressure, and cardiovascular and pulmonary disorders. Over 50% of cancer patients in both palliative and curative settings undergo radiation therapy, which can lead to various side effects. For pelvis region side effects including increased urinary and bowel frequency, rectal bleeding, and long-term shrinkage of the bladder, while hair loss, skin desquamation, tenderness, edema, hair loss, and fatigue are experiences when other parts of the body (chest, breast, head, and neck) receive radiotherapy [14]. These side effects highlight the need for adjunctive therapies that can support traditional treatments and mitigate their adverse impacts [15–18].

Dietary fiber, a nondigestible component of plant-based foods, has emerged as a potential mitigator in cancer management [19–21]. A growing body of evidence suggests that dietary fiber may play a role in reducing the risk of developing certain types of cancer and could potentially support the body's response to cancer treatment or could serve as a radiosensitizing agent to improve treatment outcomes in patients [22, 23]. The mechanisms through which dietary fiber contributes to these outcomes, however, remain an active area of research. Several studies have reported the association between dietary fiber and cancer [23–25]. In light of these considerations, a bibliometric analysis [26–30] is a powerful tool for highlighting the landscape of dietary fiber research within the context of cancer. This study is expected to reveal trends, gaps, and patterns that are not immediately apparent, informing future research directions and policymaking by systematically evaluating scientific outputs.

## 2. Experimental Section

### 2.1. Study Design

Unlike traditional literature studies that use qualitative and experimental methods, this bibliometric analysis takes a quantitative approach to assess research on dietary fiber in cancer management. The methodology examines publication metrics, temporal trends, journal distributions, and citation patterns to provide a systematic overview of the field. The analysis maps the research landscape and identifies influential works and emerging trends, analyzes the geographic and institutional distribution of research contributions, and highlights promising directions for future investigation. Through this comprehensive assessment, the study seeks to deepen understanding of dietary fiber's role in cancer management and support the development of more integrated approaches to cancer care.

### 2.2. Data Description and Collection

**Figure 1:**
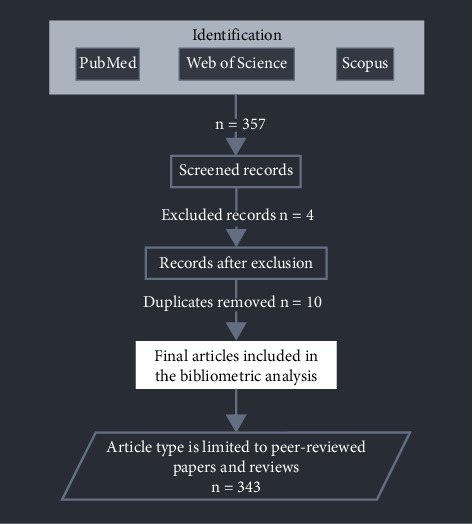
Literature search and screening process of published data on dietary fiber and cancer research from April 1999 to May 2024.

The primary sources for data collection are Web of Science, PubMed, and Scopus databases, chosen for their extensive coverage across interdisciplinary and biomedical disciplines (see Figure 1). A detailed search strategy was formulated using a combination of keywords and phrases related to “dietary fiber” and “cancer management,” including their synonyms and variations. The search query was “dietary fiber” OR “dietary fiber” OR “soluble fiber” OR “insoluble fiber” OR “fiber intake” OR “fiber intake” OR “fermentable fiber” OR “inulin” OR “psyllium” OR “pectin” “B-glucan” OR “resistant starch” OR “fructan” OR “fructose” OR “fructo-oligosaccharides” OR “oligosaccharides”) AND (“cancer management” OR “cancer treatment” OR “cancer therapy” OR “chemotherapy” OR “radiotherapy” OR “immunotherapy” OR “cancer patient care” OR “oncology” OR “nutrition”). The search was delimited to the period from April 1999 to May 2024, aiming to capture the most relevant and contemporary insights over the past 25 years.

### 2.3. Criteria for Inclusion and Exclusion of Studies

The inclusion criteria targeted peer-reviewed articles published in English from Web of Science, PubMed, and Scopus databases, focusing on the intersection between dietary fiber and cancer management. This encompasses empirical research, review articles, and meta-analyses that contribute to understanding the role of dietary fiber in the treatment, management, or care of cancer patients. Documents included were full-text publications within the specified timeframe (1999–2024).

Exclusion criteria were applied to conference abstracts and proceedings, non-peer-reviewed articles, letters to editors, book chapters, preprints, documents not in English, and publications that, despite mentioning dietary fiber or cancer, do not specifically address the role of dietary fiber in cancer management.

### 2.4. Data Cleaning

Following the initial data retrieval, a meticulous data cleaning process was undertaken. This involved the removal of duplicate entries resulting from the overlap between the databases and the refinement of search strings to weed out irrelevant articles which resulted in 343 articles included in the study (Figure 1). The relevance of each article was assessed based on its title, abstract, and keywords, ensuring a focus on the study's primary objective. The iterative optimization of search strings was instrumental in refining the dataset to a manageable and relevant collection of literature for analysis.

### 2.5. Data Analysis

For the analysis phase, the bibliometric data was analyzed using the ‘bibliometrix' R package (version 4.1.0). This package was specifically used for calculating collaboration indices, generating co-occurrence networks, and performing thematic mapping. Citation metrics were analyzed using the ‘ggplot2' package for visualization. For keyword analysis, VOSviewer software (version 1.6.18) was employed to create network visualizations and density maps. All statistical analyses were performed in R (version 4.2.0), with data preprocessing conducted using the ‘tidyverse' suite of packages.

Key metrics were derived to highlight trends in publication volume over time, identifying the most influential articles and authors through citation analysis. The study also mapped out co-authorship networks to reveal patterns of collaboration among researchers and institutions, providing insights into the community's structure. Keyword analysis further allowed the examination of research foci, emerging trends, and potential gaps in the existing literature, highlighting areas ripe for future exploration.

## 3. Results

### 3.1. Publication Trends

#### 3.1.1. Year-Wise Distribution of Publications

**Figure 2:**
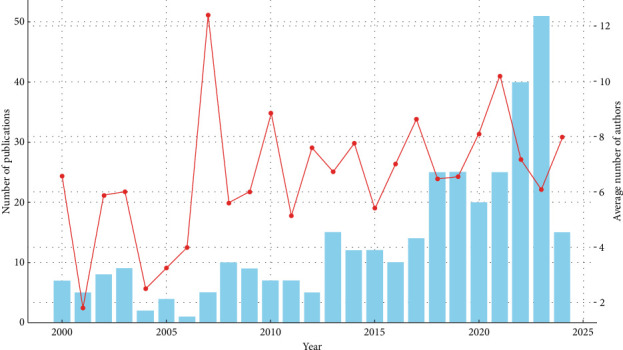
Trend analysis of scholarly publications and collaboration intensity for peer-reviewed dietary fiber and cancer research articles from 2000 to 2024.

The analysis of publication trends, as depicted in Figure 2, reveals a consistent upward trajectory in scholarly output over the observed period. No peer reviewed article was found for 1999. During the early years (2000–2010), publication activity was relatively limited, reflecting the nascent state of the research field. Significant year-to-year fluctuations during this period suggest initial interest that was not yet sustained. The subsequent mid-period (2010–2015) saw a steady growth in scholarly contributions. This period likely reflects growing awareness and recognition of the field's importance among researchers. Notably, in the recent period (2015–2023), there has been a pronounced increase in the number of publications. This surge in scholarly output, especially post-2020, suggests intensified research interest potentially driven by technological advances, policy shifts, or increased funding and collaborative opportunities.

The intensity of collaborative research, as indicated by the red line graph in Figure 2, highlights a notable evolution in the average number of authors per publication. In the early years (2000–2010), the limited collaboration, demonstrated by the relatively low average number of authors, aligns with the low overall publication output. The mid-period (2010–2015) shows a gradual rise in collaborative efforts, where research increasingly involved multiple contributors, likely due to growing interdisciplinary engagement. In the recent period (2015–2023), collaboration intensity has remained consistently high, with notable peaks in certain years. This trend signifies a transition to comprehensive, multiauthor projects, potentially enabled by the broadening of interdisciplinary collaborations, institutional partnerships, and advancements in communication technologies. The steady growth in collaboration reflects the field's maturation and the recognition that addressing complex research problems benefits from diverse expertise and multi-institutional resources.

#### 3.1.2. Geographical Distribution and Most Active Affiliations by Publications

**Figure 3:**
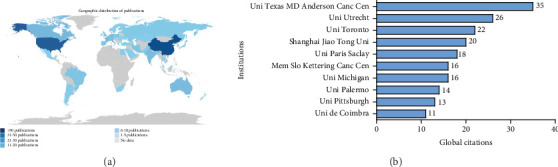
(a) Geographical distribution of most active countries and (b) top 10 most active affiliations that published peer-reviewed journals on dietary fiber and cancer research from April 1999 to May 2024.

The global distribution analysis depicted in Figure 3 reveals the concentration and reach of research activity across nations and institutions.  Figure 3a employs color gradients to represent publication output by country, with darker shades corresponding to higher volumes. This mapping demonstrates the clear dominance of the United States and China in research productivity. The substantial American contribution reflects robust research infrastructure and funding mechanisms, while the strong Chinese presence aligns with strategic national initiatives to expand research influence. Additional significant contributions emerge from the United Kingdom, Germany, and Japan, though at lower volumes than the two leading nations. The participation of regions such as Brazil and Australia indicate broader international engagement, albeit at more modest levels compared to the primary research centers.

Figure 3b presents academic institutions by their publication output in this field of research. It highlights a clear hierarchy among the top 10 contributing institutions.

University of Texas MD Anderson Cancer Center demonstrates exceptional productivity with 35 articles, establishing it as the preeminent contributor. University of Utrecht follows with 26 articles, creating a notable gap between the first and second positions. University of Toronto and Shanghai Jiao Tong University form the next tier with 22 and 20 articles, respectively.

The middle range includes University of Paris Saclay (18 articles), Memorial Sloan Kettering Cancer Center and University of Michigan (both with 16 articles), University of Palermo (14 articles), and University of Pittsburgh (13 articles). Universidade de Coimbra completes the top 10 with 11 articles.

This distribution highlights the significant role of specialized cancer research centers (MD Anderson and Memorial Sloan Kettering) alongside major research universities. The international diversity of institutions—spanning North America, Europe, and Asia—indicates the global nature of research in this domain.

The substantial lead of MD Anderson (35% more publications than Utrecht) suggests its position as a focal point for research in this field. Figure 3b also reveals a stepwise decrease in publication output across institutions, with natural groupings forming at similar publication levels.

Figure 3b further highlights institutional research output through a simple yet informative format, providing clear numerical labels that facilitate precise comparisons between the different research centers. The data suggests varying institutional capacities, priorities, or resources dedicated to research publication in this field.

### 3.2. Citation Analysis

**Figure 4:**
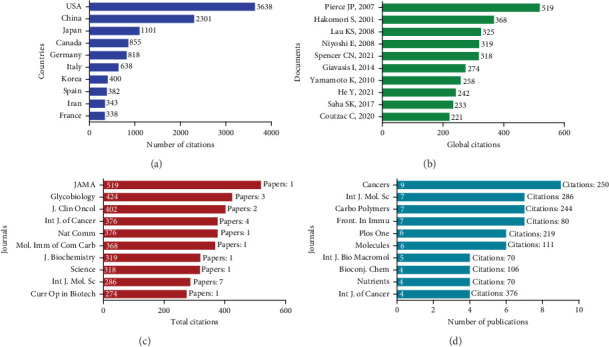
(a) Most cited countries, (b) most cited authors, (c) top 10 journals by citation, and (d) top 10 journals by the number of publications (articles) published on dietary fiber and cancer research from 1999 to 2024.

Figure 4 illustrates citation patterns among the most cited countries and individual documents, providing crucial insights into the academic influence and impact of different regions and key papers.  Figure 4a reveals the countries with the highest citation counts, offering a measure of their global impact in this field. The United States stands out prominently, far surpassing other nations with over 3600 citations. This substantial number of citations reflects the United States' research leadership and its ability to shape global academic discourse, which aligns with the dominance observed in earlier publication trends.

China emerges as the second most cited country, albeit with a significantly lower count than the United States which has over 1100 citations. The considerable gap between the two nations suggests that although China is growing in research output, the United States remains the leading authority. Japan, Germany, and Canada also feature prominently, reinforcing their status as research hubs with notable influence. Each of these countries has fostered active research communities contributing to the field's development and global scholarship. The presence of several European nations and South Korea highlights a diverse geographic spread, underlining the collaborative and international nature of research (Figure 4a).

The citation analysis revealed in Figure 4b provides fascinating insights into the research that has shaped our understanding of diet and cancer treatment. Leading the citations with over 500 global references, Pierce et al.'s groundbreaking study [31] challenged conventional wisdom about diet's role in breast cancer outcomes. Their research followed early-stage breast cancer survivors who adopted a diet extremely rich in vegetables, fruit, and fiber while maintaining low fat intake. Surprisingly, after 7.3 years of follow-up, this dietary intervention did not demonstrate the expected reduction in either additional breast cancer events or mortality rates.

Hakomori's research [32] opened new frontiers in cancer immunotherapy by exploring tumor-associated carbohydrate antigens (TACAs) as targets for anticancer vaccines. Their clinical trials demonstrated promising results across multiple cancer types: STn vaccines showed potential for breast cancer treatment, while GM2 and GD3 proved beneficial for melanoma cases, and globo-H showed promise in treating prostate cancer. The study established crucial criteria for successful vaccine development, emphasizing the importance of high antigen expression on tumor cells and robust antibody production. While they also explored innovative approaches using idiotypic anticarbohydrate antibodies and peptide mimetics of TACAs, these methods remain in early stages of development but offer potential solutions to the challenge of weak TACA immunogenicity.

Spencer et al.'s research [33] revealed an important connection between dietary fiber and cancer immunotherapy outcomes. Their findings demonstrated that patients receiving immune checkpoint blockade (ICB) treatment showed improved progression-free survival when maintaining high fiber intake. Notably, this benefit was particularly pronounced in patients who consumed sufficient fiber while avoiding probiotic supplements, suggesting a complex interaction between diet, gut microbiota, and cancer treatment efficacy.

Giavasis [34] contributed valuable insights into the therapeutic potential of fungal compounds, particularly focusing on polysaccharides like *β*-glucans and heteropolysaccharides. Their research traced how these compounds evolved from traditional Asian medicine into modern pharmaceutical applications. The study highlighted these substances' versatility in medical applications, specifically their effectiveness as antitumor agents, immune system stimulants, and prophylactic treatments. This work effectively bridged the gap between traditional medical knowledge and contemporary pharmaceutical development.

Figure 4c presents the most influential (cited) journals in dietary fiber and cancer research. Notably, the Journal of the American Medical Association (JAMA) dominates the citation landscape with 519 citations from just one paper from the dataset, demonstrating extraordinary impact. Similarly, Glycobiology and Journal of Clinical Oncology show significant influence with 424 and 402 citations, respectively.

In contrast, journals such as Cancers, International Journal of Molecular Sciences, Carbohydrate Polymers, and Frontiers in Immunology lead in publication volume, indicating their role as primary venues for ongoing research in this field (Figure 4d). Furthermore, the minimal overlap between the two panels (Figure 4c,d) suggests researchers employ different publication strategies depending on their goals. Breakthrough findings often target prestigious journals with wide readership, whereas more specialized research appears in field-specific journals.

Additionally, the diversity of journals across both panels reflects the multidisciplinary nature of using nutritional intervention including dietary fiber and cancer research, spanning from general medicine to biochemistry, oncology, and molecular sciences. Interestingly, the International Journal of Molecular Sciences appears among leaders in both metrics, suggesting its dual role as a popular venue with substantial impact.

The implication of this is significant for research strategy and scholarly communication. Researchers seeking to maximize visibility, and impact might prioritize submitting high-quality work to citation-dominant journals like Journal of the American Medical Association or Glycobiology, while those needing to build publication volume quickly might target journals like Cancers. For institutions evaluating research performance, Figure 4c demonstrates the importance of considering both metrics rather than focusing solely on publication counts. The substantial disparity between publication output and citation impact across journals also suggests fundamentally different roles within the scholarly ecosystem—some journals serve primarily as venues for disseminating new findings, while others function as platforms that significantly amplify research influence through citations.

### 3.3. Keyword and Topic Trend Analysis

**Figure 5:**
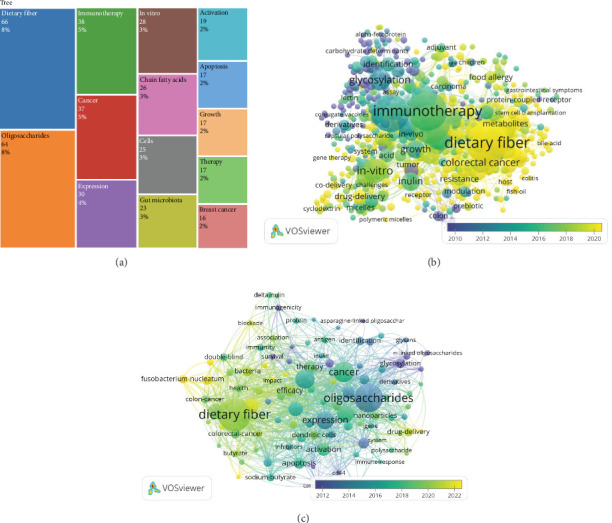
(a) Top 14 most frequent author keywords from the peer-reviewed articles on dietary fiber and cancer research from 1999 to 2024, (b) temporal trends in keyword co-occurrence in dietary fiber and cancer research from 2010 to 2020, and (c) expanded keyword co-occurrence network in dietary fiber and cancer research (2012–2022).

The analysis of Figure 5 depicts the most frequent author keywords and reveals several dominant research themes. The analysis of keywords, as depicted in Figure 5a, reveals the dominant research themes in the field. ‘Dietary fiber' emerges as the most frequent keyword with 66 occurrences (8% of total keywords), followed closely by ‘oligosaccharides' with 64 occurrences (8%). These two major themes reflect the core focus of the research field. ‘Immunotherapy' and ‘cancer' represent the next tier of frequently used keywords with 38 (5%) and 37 (5%) occurrences, respectively, indicating strong research interest in therapeutic applications.

The visualization also highlights several interconnected research areas: ‘in-vitro' (28 occurrences, 3%), ‘chain fatty-acids' (26 occurrences, 3%), and ‘cells' (25 occurrences, 3%) suggest significant laboratory-based experimental work. ‘Expression' (30 occurrences, 4%) and ‘gut microbiota' (23 occurrences, 3%) indicate a growing interest in molecular mechanisms and microbiome research. More specific disease-focused terms appear as well, such as ‘breast-cancer' (16 occurrences, 2%), while process-oriented terms like ‘activation' (19 occurrences, 2%), ‘apoptosis' (17 occurrences, 2%), and ‘growth' (17 occurrences, 2%) reflect the emphasis on understanding biological mechanisms.

#### 3.3.1. Co-Occurrence Network of Keywords

The co-occurrence network in Figure 5b illustrates a comprehensive relationship between the most prevalent research themes. Two big primary topics, “dietary fiber” and “immunotherapy,” are situated centrally in the network, indicating strong connections with various other keywords. “Dietary fiber,” marked predominantly in yellow and green shades, reflects its recent rise in prominence from 2018 onward. This keyword is closely associated with related themes like “colorectal cancer,” “inulin,” and “growth,” underscoring its critical role in metabolic health and cancer research.

Similarly, “immunotherapy,” also marked in yellow and green, shows strong associations with “in-vitro,” “drug delivery,” “resistance,” and “modulation,” highlighting the current interest in experimental and therapeutic strategies to enhance immune responses against various diseases.

The overlay color gradient provides an informative timeline, ranging from purple/blue (earlier prominence) to green/yellow (more recent interest). The yellow and green clusters represent the most current research themes, indicating a heightened focus on “dietary fiber,” “immunotherapy,” “glycosylation,” and “colorectal cancer” since 2016. These terms are linked with emerging interdisciplinary topics like “food allergy,” “resistance,” and “polymeric micelles,” demonstrating recent growth in gut health and drug delivery systems.

Darker colors (purple/blue) represent research topics that were prominent before 2016, such as “identification,” “adjuvant,” and “growth.” These foundational themes laid the groundwork for newer concepts like advanced therapeutics and disease modulation.

Figure 5b also provides a valuable snapshot of the evolving research landscape, illustrating how central themes like dietary fiber and immunotherapy are interconnected with emerging and foundational concepts. The color gradient timeline highlights evolving interests over time, offering a concise overview of the field's thematic clusters and how past research has informed recent trends. This network is crucial for guiding researchers toward new collaboration opportunities and helping them identify the most relevant emerging themes.

Figure 5c shows a detailed co-occurrence network of keywords plus, which expands on previous analyses by incorporating additional, author-provided keywords. This visualization helps reveal the relationships and emerging trends across a broader spectrum of themes. Two primary themes stand out prominently in this network: “dietary fiber” and “oligosaccharides.” Both terms occupy central positions and are highlighted with larger font sizes, indicating their significance and interconnectedness with other keywords. “Dietary fiber,” predominantly shaded in green and yellow, signifies recent research interest (2018–2022) and links with related terms like “colorectal cancer,” “butyrate,” and “health.” This centrality highlights the critical role dietary fiber plays in gastrointestinal and metabolic health.

“Oligosaccharides” is marked similarly in green and yellow, reflecting recent interest. It is closely linked to terms like “bacteria,” “glycosylation,” and “drug delivery,” highlighting its relevance in gut health, immune modulation, and therapeutic approaches.

The color gradient timeline ranges from purple (earlier prominence, pre-2014) to yellow (recent prominence, 2020–2022), revealing evolving research interests. Keywords like “dietary fiber,” “oligosaccharides,” and “therapy” appear in green/yellow shades, indicating increasing focus since 2018. Their associations with emerging themes like “drug delivery,” “health,” and “association” suggest interdisciplinary growth. Darker colors (purple/blue) represent foundational topics that gained prominence before 2014, such as “bacteria,” “delta-inulin,” and “fusobacterium-nucleatum.” Their enduring relevance forms the groundwork for recent research. Central themes like dietary fiber and oligosaccharides remain essential while other emerging topics gain prominence (Figure 5c).

### 3.4. Thematic Areas

**Figure 6:**
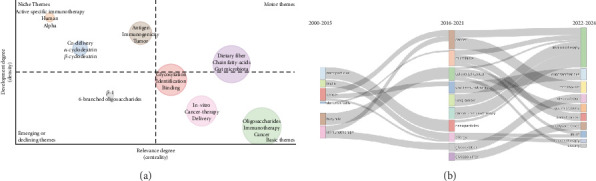
(a) Strategic quadrant analysis of research themes in dietary fiber and cancer peer reviewed published articles from 2000 to 2024 and (b) temporal transition of key research themes in dietary fiber and cancer research from 2000 to 2024.

The thematic map in Figure 6 divides research topics into four quadrants based on relevance and development, offering valuable insights into the maturity and impact of various themes.

In the upper-right quadrant (Motor Themes), we find “dietary fiber,” “chain fatty-acids,” and “gut microbiota.” These themes are well-developed and highly relevant, signifying their central role in shaping current research. Their positioning indicates strong development and high centrality to the field.

The upper-left quadrant (Niche Themes) features topics like “active specific immunotherapy,” “human alpha,” and “beta-cyclodextrin.” Despite their high level of development, these themes have lower relevance to the broader field. This specialized focus suggests their significance is confined to specific subfields rather than across a wide spectrum of research interests.

In the lower-left quadrant (Emerging or Declining Themes), topics like “beta-1” and “6-branched oligosaccharides” are less relevant and underdeveloped, hinting at either an emerging status or a gradual decline. Their relatively low relevance indicates that they are currently niche areas, possibly gaining traction or becoming obsolete over time.

The lower-right quadrant (Basic Themes) includes “glycosylation,” “identification,” and “binding.” These themes show high relevance but lower development, suggesting they serve as fundamental concepts underpinning more complex research areas in the field.

### 3.5. Evolution of Research Themes Over Time

Figure 6b illustrates the evolution of research themes over time, specifically comparing the periods of 2000–2015, 2016–2021, and 2022–2024. This visualization provides insights into how key themes have emerged, persisted, or evolved across these time frames.

In the first period (2000–2015), notable themes included “cancer,” “nanoparticles,” “dendritic cells,” “butyrate,” “inulin,” and “immunotherapy.” These topics represented a diverse range of interests, with “cancer” leading the field as a focal point. “Nanoparticles” and “dendritic cells” indicated a strong interest in innovative therapeutic delivery mechanisms and immune cell studies [35].

Moving to the next period (2016–2021), several themes persisted and evolved, while others shifted or emerged. “Cancer” remained prominent and expanded into subcategories like “colorectal cancer,” “lung cancer,” and “oral immunotherapy.” Newer themes like “microbiota” and “allergy” emerged, signifying a growing focus on the microbiome and its health implications. “Glycosylation” gained prominence, suggesting an increased interest in biochemical processes relevant to cancer research [36].

In the latest period (2022–2024), several core themes have persisted or expanded in relevance. “Immunotherapy,” “inulin,” and “gut microbiota” remain prominent, underscoring the enduring interest in immune-based treatments, carbohydrate biology, and microbiome research. Emerging themes like “dietary,” “polysaccharides,” and “cancer therapy” reflect the importance of nutritional strategies and biochemical processes in disease management. “Glycosylation,” “immunotherapy,” and “inulin” continue to play key roles, suggesting continuity in research focus [35–37].

Figure 6b effectively demonstrates how research priorities shift and evolve. Cancer-related themes have consistently remained central, with subdomains developing over the years. The rising prominence of themes like “microbiota” and “oligosaccharides” signals an expanding interest in gut health and therapeutic innovation. The persistence of topics like “immunotherapy” and “glycosylation” indicates their continued relevance across these periods. Such a diagram helps researchers identify long-standing trends and anticipate emerging directions, guiding them in strategic planning and collaboration.

## 4. Discussion

The bibliometric findings from this study offer a comprehensive overview of the research landscape's evolution, highlighting key trends, thematic shifts, and geographical distribution patterns. The increasing number of publications over time indicates growing scholarly interest and recognition of the field's importance. In particular, the rise in publication volume over the past decade, especially concerning interdisciplinary research areas like immunotherapy, gut health, and dietary fiber, suggests a paradigm shift in how these topics are perceived [38, 39]. This shift highlights the vital role of technological advancements and collaborative networks in driving research forward.

In this study, key themes, such as the relationship between dietary fiber and gut health and their impact on colorectal cancer, have consistently dominated the field. The focus on specific cancer types demonstrates a targeted approach to understanding dietary and immunological influences on the most prevalent forms of cancer [33]. In recent decades, there has been increasing evidence of the importance of lifestyle (such as exercise, diet, and sleep) on human health and disease development. These lifestyles can contribute to chronic ill health such as cancer, diabetes, and cardiovascular diseases [40].

Diet has been reported as a major key modulator of the gut microbiota which has been associated with human health and the development of disease conditions [41]. The intestinal microbiota ferments undigestible carbohydrates from food (dietary fiber) to produce metabolites such as short-chain fatty acids, including acetate, butyrate, and propionate, and neurotransmitters which are beneficial to host health [42, 43]. This bibliometric analysis also showed the dominance of key themes like metabolites, butyrate, and short-chain fatty acids in dietary fiber studies. Previous studies have emphasized the interplay between diet, gut microbiota, gastrointestinal health, and cancer therapies. Butyrate has been reported to be used as an energy source by colonocytes [44, 45]. Also, high abundance of bacteria genera including *Bifidobacterium* and *Lactobacillus* have been strongly linked with anti-inflammatory and antitumorigenic properties, suggesting a potential association between dietary fiber, microbiota composition, and survival benefits to patients [46, 47]. Significant progress has been achieved in the identification of microbial populations and their impact on host health, thanks to technical advancements such as 16S rRNA gene sequencing, shotgun sequencing, metabolomics, and other analytical tools [41–43].

Similarly, this study has shown increasing interest in immunotherapy which indicates a transition from purely preventive strategies to therapeutic applications, such as immune-based treatments. This strategy harnesses the responses from the immune system in cancer treatment and management [48]. Immunotherapy employs the use of several agents including chimeric antigen receptor T-cell therapy, cancer vaccines, and the immune checkpoint inhibitors which are gaining popularity. Dietary fiber supplementation has been found to lead to reduction in T-cells which may be mediated by gut-derived metabolites, consequently altering immune microenvironment thereby influencing T-cell modulation, tumor immune evasion, and systemic health [49, 50]. Immunotherapy has shown some promising results and provides a practical approach to improve treatment outcomes and increase the overall survival rate of people with cancer [51].

From 2010 onwards, an upward trajectory in scholarly output was observed for studies on the importance of dietary fiber which is associated with growing interest in dietary studies among researchers. This surge in publications suggests intensified research interest potentially driven by technological advances, policy shifts, or increased funding and collaborative opportunities. Studies such as Pierce et al. [31] lead with over 500 global citations, emphasizing the lasting impact of this work on subsequent research. Other highly cited documents include Hakomori [32], Spencer et al. [33], and Giavasis [34].

Additionally, the citation count and publication output from the current study also showed the relevance of countries including the United States and China in the advancement of dietary fiber and cancer studies. This reflects strong research environments, sustained funding and strategic institutional support. For instance, in 2024, the Chinese Government pledged $52 billion to support the development of Science and Technology which is a 10% increase from the previous year [52] while the US Government budgeted $209 billion for Research and Development, a 4% increase from 2023 [53]. Other regions including Australia, Canada, Germany, Japan, and the United Kingdom also contribute significantly, indicating a global collaborative network that enriches the research landscape.

The analysis of co-authorship networks emphasizes the importance of collaboration on a multidisciplinary and multi-institutional level to increase research quality and depth, enabling a comprehensive exploration of complex themes. The cross-pollination of ideas across institutions and disciplines drives innovation, enhances the breadth of research, and helps translate findings into practical health solutions [54]. These findings provide a strategic roadmap for researchers and institutions, offering a coherent understanding of how the field has evolved. Scholars can strategically guide their future research, ensuring continued progress in this dynamic and impactful area by aligning research efforts with these emerging trends, identifying new collaboration opportunities, and recognizing foundational themes.

## 5. Future Directions and Research Gaps

The bibliometric analysis has identified several critical areas requiring further investigation. While butyrate emerges as a key theme in our keyword analysis, more research is needed to fully understand its impact on tumor biology across different cancer types. Future studies should examine how varying fiber types, and their fermentation products differently affect specific cancers, potentially leading to personalized dietary interventions.

The publication trends reveal a need for well-designed clinical trials that evaluate how different fiber types influence therapeutic outcomes. These trials should assess fiber's synergistic effects with standard treatments and examine dosage optimization for various cancer stages. The institutional analysis suggests a need for stronger multidisciplinary collaboration—particularly among oncologists, nutritionists, and microbiome researchers—to bridge the gap between laboratory findings and clinical applications.

The co-occurrence network analysis highlights opportunities for integrating dietary interventions with conventional cancer treatments. This integration requires systematic evaluation through controlled trials and careful consideration of timing and sequencing of dietary interventions alongside standard therapies.

The rising prominence of the gut microbiota as a key research theme necessitates further investigation into its relationship with dietary fiber. Understanding how gut microbiota mediates systemic inflammation, immune responses, and cancer metastasis is critical. Research should delve into the mechanistic pathways by which dietary fibers modulate immune responses within the tumor microenvironment, providing novel therapeutic strategies that leverage the gut–immune system relationship.

Progress in this field depends on integrating insights across multiple disciplines. Nutritional science, molecular biology, microbiology, and clinical oncology must converge to decode complex interactions between diet and cancer biology. Collaborative efforts between researchers and clinicians can transform bench-side discoveries into practical applications. Leveraging technological advances in computational biology, shotgun sequencing, and whole-genome sequencing will be crucial in modeling these interactions, potentially uncovering personalized nutritional strategies for cancer prevention and management.

The geographical distribution of research output reveals that the field remains heavily concentrated in specific regions. This concentration presents a need for a more granular exploration of the effects of dietary patterns on cancer across diverse populations. Understanding how cultural dietary habits, lifestyle factors, and genetic backgrounds influence gut health and cancer susceptibility will facilitate personalized dietary recommendations. Such targeted research can help identify risk factors or protective elements relevant to different populations, leading to more equitable health outcomes.

Emerging applications, such as the use of nanoparticles and polymeric micelles in drug delivery, offer promising yet underexplored areas for cancer therapy. Additionally, research into the synergistic effects of dietary fiber with other nutrients and their impact on therapeutic outcomes should be prioritized. Exploring these applications could pave the way for innovative strategies that maximize the benefits of personalized treatments. Addressing these gaps and exploring future directions will provide crucial insights into dietary fiber's multifaceted role in cancer therapy, helping to shape more effective, patient-centered strategies for prevention and management.

## 6. Conclusions

This bibliometric analysis provides a comprehensive exploration of the research landscape in the fields of dietary fiber, cancer therapy, gut health, and immunotherapy. The analysis has revealed a steady growth in publications over the years, which shows the increasing recognition of these themes' significance. The findings highlight key themes, such as dietary fiber, gut microbiota, immunotherapy, and cancer therapy, that have dominated the research landscape while revealing the nuanced relationships between these themes and emerging concepts.

The study has identified the significant role of collaboration and interdisciplinary research in advancing the field, as demonstrated by the increasing co-authorship trends and geographical distribution of research activity. The thematic evolution indicates a paradigm shift towards innovative, personalized, and multidisciplinary approaches that incorporate insights from nutrition science, molecular biology, microbiology, and clinical oncology.

Despite the progress, this analysis has also identified several research gaps that present future opportunities. A deeper exploration into the nuanced impacts of dietary fiber on cancer progression, personalized nutrition strategies, and the role of gut microbiota and immune modulation is crucial. Additionally, integrating technological advancements in computational biology will be vital in understanding these complex relationships.

This analysis provides researchers, policymakers, and clinicians with valuable insights to guide future investigations and strategic collaborations. The academic community can shape more effective, equitable, and comprehensive strategies for cancer prevention and therapy, ultimately improving patient outcomes by understanding the evolving research themes and addressing the identified gaps.

## Data Availability

Data are available upon reasonable request from the corresponding author.

## Nomenclature

Bioconj. Chem: Bioconjugate Chemistry
Carbo Polymers: Carbohydrate Polymers
Curr Op in Biotech: Current Opinion in Biotechnology
Front. In Immu: Frontiers in Immunology
ISAPP: International Scientific Association for Probiotics and Prebiotics
JAMA: Journal of the American Medical Association
J. Biochemistry: Journal of Biochemistry
J. Clin Oncol: Journal of Clinical Oncology
Int J. Bio Macromol: International Journal of Biological Macromolecules
Int J. of Cancer: International Journal of Cancer
Int J. Mol. Sc: International Journal of Molecular Sciences
Mol. Imm of Com Carb: Molecular Immunology of Complex Carbohydrates
Nat Comm: Nature Communications
PLOS ONE: Public Library of Science One
rRNA: ribosomal ribonucleic acid
TACAs: tumor-associated carbohydrate antigens
VOSviewer: Visualization of Similarities viewer

## References

[1] Jin L., Lamster I., Greenspan J., Pitts N., Scully C., Warnakulasuriya S. (2016). Global Burden of Oral Diseases: Emerging Concepts, Management and Interplay With Systemic Health. *Oral Diseases*.

[2] Brown M. L., Lipscomb J., Snyder C. (2001). The Burden of Illness of Cancer: Economic Cost and Quality of Life. *Annual Review of Public Health*.

[3] Murphy B. A., Ridner S., Wells N., Dietrich M. (2007). Quality of Life Research in Head and Neck Cancer: A Review of the Current State of the Science. *Critical Reviews in Oncology/Hematology*.

[4] Bray F., Ferlay J., Soerjomataram I., Siegel R. L., Torre L. A., Jemal A. (2018). Global Cancer Statistics 2018: GLOBOCAN Estimates of Incidence and Mortality Worldwide for 36 Cancers in 185 Countries. *CA: A Cancer Journal for Clinicians*.

[5] Torre L. A., Siegel R. L., Ward E. M., Jemal A. (2016). Global Cancer Incidence and Mortality Rates and Trends—An Update. *Cancer Epidemiology, Biomarkers & Prevention*.

[6] Siegel R. L., Miller K. D., Jemal A. (2019). Cancer Statistics, 2019. *CA: A Cancer Journal for Clinicians*.

[7] Sung H., Ferlay J., Siegel R. L. (2021). Global Cancer Statistics 2020: GLOBOCAN Estimates of Incidence and Mortality Worldwide for 36 Cancers in 185 Countries. *CA: A Cancer Journal for Clinicians*.

[8] Keum N., Giovannucci E. (2019). Global Burden of Colorectal Cancer: Emerging Trends, Risk Factors and Prevention Strategies. *Nature Reviews. Gastroenterology & Hepatology*.

[9] Adam A., Kenny L. M. (2015). Interventional Oncology in Multidisciplinary Cancer Treatment in the 21^st^ Century. *Nature Reviews Clinical Oncology*.

[10] Reed M. (2009). Principles of Cancer Treatment by Surgery. *Surgery (Oxford)*.

[11] Hartshorn C. M., Bradbury M. S., Lanza G. M. (2018). Nanotechnology Strategies to Advance Outcomes in Clinical Cancer Care. *ACS Nano*.

[12] Du J., Tang X. (2014). Natural Products Against Cancer: A Comprehensive Bibliometric Study of the Research Projects, Publications, Patents and Drugs. *Journal of Cancer Research and Therapeutics*.

[13] Basmadjian C., Zhao Q., Bentouhami E. (2014). Cancer Wars: Natural Products Strike Back. *Frontiers in Chemistry*.

[14] Pilleron S., Alqurini N., Ferlay J. (2022). International Trends in Cancer Incidence in Middle-Aged and Older Adults in 44 Countries. *Journal of Geriatric Oncology*.

[15] Markiewicz D. A., Schultz D. J., Haas J. A. (1996). The Effects of Sequence and Type of Chemotherapy and Radiation Therapy on Cosmesis and Complications After Breast Conservation Therapy. *International Journal of Radiation Oncology • Biology • Physics*.

[16] Albano D., Benenati M., Bruno A. (2021). Imaging Side Effects and Complications of Chemotherapy and Radiation Therapy: A Pictorial Review From Head to Toe. *Insights into Imaging*.

[17] Wargo J. A., Reuben A., Cooper Z. A., Oh K. S., Sullivan R. J. (2015). Immune Effects of Chemotherapy, Radiation, and Targeted Therapy and Opportunities for Combination With Immunotherapy. *Seminars in Oncology*.

[18] Meistrich M. L. (2013). Effects of Chemotherapy and Radiotherapy on Spermatogenesis in Humans. *Fertility and Sterility*.

[19] McClements D. J., Grossmann L. (2022). Nutritional and Health Aspects. *Next-Generation Plant-Based Foods*.

[20] Cencic A., Chingwaru W. (2010). The Role of Functional Foods, Nutraceuticals, and Food Supplements in Intestinal Health. *Nutrients*.

[21] Didinger C., Foster M. T., Bunning M., Thompson H. J. (2022). Nutrition and Human Health Benefits of Dry Beans and Other Pulses. *Dry Beans and Pulses*.

[22] Eaton S. E., Kaczmarek J., Mahmood D. (2022). Exploiting Dietary Fibre and the Gut Microbiota in Pelvic Radiotherapy Patients. *British Journal of Cancer*.

[23] Then C. K., Paillas S., Moomin A. (2024). Dietary Fibre Supplementation Enhances Radiotherapy Tumour Control and Alleviates Intestinal Radiation Toxicity. *Microbiome*.

[24] Keizman D., Frenkel M., Peer A. (2021). Modified Citrus Pectin Treatment in Non-Metastatic Biochemically Relapsed Prostate Cancer: Results of a Prospective Phase II Study. *Nutrients*.

[25] Li Y., Elmén L., Segota I. (2020). Prebiotic-Induced Anti-Tumor Immunity Attenuates Tumor Growth. *Cell Reports*.

[26] Kostoff R. N., Toothman D. R., Eberhart H. J., Humenik J. A. (2001). Text Mining Using Database Tomography and Bibliometrics: A Review. *Technological Forecasting and Social Change*.

[27] Moosavi J., Naeni L. M., Fathollahi-Fard A. M., Fiore U. (2021). Blockchain in Supply Chain Management: A Review, Bibliometric, and Network Analysis. *Environmental Science and Pollution Research*.

[28] Yu D., Xu Z., Pedrycz W. (2020). Bibliometric Analysis of Rough Sets Research. *Applied Soft Computing*.

[29] Gawusu S., Mensah R. A., Das O. (2022). Exploring Distributed Energy Generation for Sustainable Development: A Data Mining Approach. *Journal of Energy Storage*.

[30] Gawusu S., Zhang X., Ahmed A. (2022). Renewable Energy Sources From the Perspective of Blockchain Integration: From Theory to Application. *Sustainable Energy Technologies and Assessments*.

[31] Pierce J. P., Natarajan L., Caan B. J. (2007). Influence of a Diet Very High in Vegetables, Fruit, and Fiber and Low in Fat on Prognosis Following Treatment for Breast Cancer. *Jama*.

[32] Hakomori S. (2001). *Tumor-Associated Carbohydrate Antigens Defining Tumor Malignancy: Basis for Development of Anti-Cancer Vaccines. The Molecular Immunology of Complex Carbohydrates —2*.

[33] Spencer C. N., McQuade J. L., Gopalakrishnan V. (2021). Dietary Fiber and Probiotics Influence the Gut Microbiome and Melanoma Immunotherapy Response. *Science*.

[34] Giavasis I. (2014). Bioactive Fungal Polysaccharides as Potential Functional Ingredients in Food and Nutraceuticals. *Current Opinion in Biotechnology*.

[35] Liu C. G., Han Y. H., Kankala R. K., Wang S. B., Chen A. Z. (2020). Subcellular Performance of Nanoparticles in Cancer Therapy. *International Journal of Nanomedicine*.

[36] Liu D., Wang Y., Li X. (2023). Participation of Protein Metabolism in Cancer Progression and Its Potential Targeting for the Management of Cancer. *Amino Acids*.

[37] Hönigova K., Navratil J., Peltanova B., Polanska H. H., Raudenska M., Masarik M. (2022). Metabolic Tricks of Cancer Cells. *Biochimica et Biophysica Acta (BBA)-Reviews on Cancer*.

[38] Ogino S., Nowak J. A., Hamada T., Milner D. A., Nishihara R. (2019). Insights Into Pathogenic Interactions Among Environment, Host, and Tumor at the Crossroads of Molecular Pathology and Epidemiology. *Annual Review of Pathology: Mechanisms of Disease*.

[39] Anagnostou A., Lieberman J., Greenhawt M. (2023). The Future of Food Allergy: Challenging Existing Paradigms of Clinical Practice. *Allergy*.

[40] Barber T. M., Kabisch S., Pfeiffer A. F. H., Weickert M. O. (2020). The Health Benefits of Dietary Fibre. *Nutrients*.

[41] Bester A., O’Brien M., Cotter P., Dam S., Civai C. (2023). Shotgun Metagenomic Sequencing Revealed the Prebiotic Potential of a Fruit Juice Drink With Fermentable Fibres in Healthy Humans. *Food*.

[42] Silva M., Cueva C., Alba C. (2022). Gut Microbiome-Modulating Properties of a Polyphenol-Enriched Dietary Supplement Comprised of Hibiscus and Lemon Verbena Extracts. Monitoring of Phenolic Metabolites. *Journal of Functional Foods*.

[43] Chen Y., Xu J., Chen Y. (2021). Regulation of Neurotransmitters by the Gut Microbiota and Effects on Cognition in Neurological Disorders. *Nutrients*.

[44] Sanchez H. N., Moroney J. B., Gan H. (2020). B Cell-Intrinsic Epigenetic Modulation of Antibody Responses by Dietary Fiber-Derived Short-Chain Fatty Acids. *Nature Communications*.

[45] Duan H., Wang L., Huangfu M., Li H. (2023). The Impact of Microbiota-Derived Short-Chain Fatty Acids on Macrophage Activities in Disease: Mechanisms and Therapeutic Potentials. *Biomedicine & Pharmacotherapy*.

[46] Gavzy S. J., Kensiski A., Lee Z. L., Mongodin E. F., Ma B., Bromberg J. S. (2023). *Bifidobacterium* Mechanisms of Immune Modulation and Tolerance. *Gut Microbes*.

[47] Heydari Z., Rahaie M., Alizadeh A. M. (2019). Different Anti-Inflammatory Effects of *Lactobacillus acidophilus* and *Bifidobactrum bifidioum* in Hepatocellular Carcinoma Cancer Mouse Through Impact on MicroRNAs and Their Target Genes. *Journal of Nutrition & Intermediary Metabolism*.

[48] Gonzalez H., Hagerling C., Werb Z. (2018). Roles of the Immune System in Cancer: From Tumor Initiation to Metastatic Progression. *Genes & Development*.

[49] Voena C., Chiarle R. (2016). Advances in Cancer Immunology and Cancer Immunotherapy. *Discovery Medicine*.

[50] Mellman I., Coukos G., Dranoff G. (2011). Cancer Immunotherapy Comes of Age. *Nature*.

[51] Ling S. P., Ming L. C., Dhaliwal J. S. (2022). Role of Immunotherapy in the Treatment of Cancer: A Systematic Review. *Cancers*.

[52] Mallapaty S. (2024). China Promises More Money for Science in 2024. *Nature*.

[53] Sargent J. F. (2022). *Federal Research and Development (R&D) Funding: FY2024*.

[54] Berardi R., Morgese F., Rinaldi S. (2020). Benefits and Limitations of a Multidisciplinary Approach in Cancer Patient Management. *Cancer Management and Research*.

